# Vitamin D: A Review on Its Effects on Muscle Strength, the Risk of Fall, and Frailty

**DOI:** 10.1155/2015/953241

**Published:** 2015-04-27

**Authors:** Matthieu Halfon, Olivier Phan, Daniel Teta

**Affiliations:** Service of Nephrology, Department of Medicine, Centre Hospitalier Universitaire Vaudois (CHUV), Lausanne, Switzerland

## Abstract

Vitamin D is the main hormone of bone metabolism. However, the ubiquitary nature of vitamin D receptor (VDR) suggests potential for widespread effects, which has led to new research exploring the effects of vitamin D on a variety of tissues, especially in the skeletal muscle. *In vitro* studies have shown that the active form of vitamin D, calcitriol, acts in myocytes through genomic effects involving VDR activation in the cell nucleus to drive cellular differentiation and proliferation. A putative transmembrane receptor may be responsible for nongenomic effects leading to rapid influx of calcium within muscle cells. Hypovitaminosis D is consistently associated with decrease in muscle function and performance and increase in disability. On the contrary, vitamin D supplementation has been shown to improve muscle strength and gait in different settings, especially in elderly patients. Despite some controversies in the interpretation of meta-analysis, a reduced risk of falls has been attributed to vitamin D supplementation due to direct effects on muscle cells. Finally, a low vitamin D status is consistently associated with the frail phenotype. This is why many authorities recommend vitamin D supplementation in the frail patient.

## 1. Introduction

Vitamin D is the main hormone regulating calcium phosphate homeostasis and mineral bone metabolism. The discovery that a variety of tissues can express vitamin D receptor (VDR) has opened new ways of research related to vitamin D biological effects and molecular pathways [1–3]. There is evidence that vitamin D is implicated in the regulation of the immune system, the cardiovascular system, oncogenesis [4], and cognitive functions [5].

Loss of muscle mass and frailty are prevalent in many chronic diseases such as chronic obstructive pulmonary disease, cardiac insufficiency, cancer, and chronic kidney disease (CKD) [6].

Vitamin D deficiency is indeed extremely frequent in the above diseases. More than 3 decades ago, the clinical observation that patients with rickets and osteomalacia displayed proximal myopathy suggested a direct link between hypovitaminosis D and muscle function [7]. Recent evidence has confirmed that vitamin D may modulate muscle growth. In this review, we will specifically address the effect of vitamin D on skeletal muscles and its clinical implications, especially frailty and the risk of fall.

## 2. Methods

This is a review article not intended to meet the full range of criteria required for a systematic review. However, we used a rigorous methodology for the selection of the material presented here. In October 2014, we performed a comprehensive literature search in the bibliographic database “Pubmed,” looking at studies discussing the following topics: “hypovitaminosis D and physical performance: observational studies,” “can vitamin D supplementation improve muscle function?,” “relationship between vitamin D status, muscle and falls,” and “link between vitamin D and frailty.” The study period considered was from January 1, 2000, to September 30, 2014. Keywords used were “vitamin D,” “muscle,” “strength,” “fall,” “frailty,” “risk,” “chronic kidney disease,” “supplementation,” “randomized controlled trial,” “review,” “systematic review,” and “meta analysis.” Only articles in English were considered. From the studies identified in this way, we selected only observational trials, randomized control trials (RCTs), and meta-analysis. Case reports and series were excluded. In studies testing vitamin D supplementation, the dose of vitamin D should have been specified, physical performance/gait should have been reported by objective measurements, and blood level of vitamin D should have been reported. In studies regarding frailty, the terminology of frailty should have been defined by objective criteria. Finally, we did not include the totality of studies meeting the above criteria, in order to prevent redundancies. Studies confirming results from previous relevant studies and providing similar conclusions were voluntarily not cited.

## 3. Vitamin D Metabolism

Vitamin D metabolism is orchestrated by the skin, the liver, and the kidney. The role of sun exposure is instrumental since UVB-induced vitamin D3 production in the skin accounts for 80–90% of vitamin D formation, whereas nutritional intake (fatty fish, eggs, fortified milk, and plants) only accounts for 10–20% of vitamin D3 provision. UVB converts 7-dehydrocholesterol to previtamin D which is then converted to cholecalciferol (or vitamin D3). Cholecalciferol subsequently binds to vitamin D binding globulin and this complex is transported to the liver where it is hydroxylated in 25-hydroxyvitamin D3 (or 25(OH) D3), the major circulating form. 25-Hydroxyvitamin D3 undergoes a final hydroxylation in the kidney proximal tubule in order to produce 1,25 dihydroxyvitamin D3 or calcitriol, the biologically active form [8]. The 1-hydroxylation is stimulated by the parathyroid hormone (PTH) and inhibited by the Fibroblast Growth Factor 23 (FGF-23). Calcitriol interacts with vitamin D receptor (VDR) in the cell nucleus to mediate biological effects through activation of calcium channels. Vitamin D synthesis depends on environmental factors such as sunlight exposure and sun cream application [9] and biologic factors such as skin's pigmentation and kidney function. In elderly people, dietary vitamin D (vitamin D2 from plants and vitamin D3 from animals) may become the major source of vitamin D3 because of reduced 7-dehydrocholesterol concentration [10] and impaired hydroxylation in the liver and the kidney [11]. This is why the relevance of vitamin D status is highlighted in this population. This is particularly true for the elderly frail patient clinically characterized by a low nutritional intake and muscle loss. Whether vitamin D deficiency may aggravate muscle function and frailty is thus a very important question.

## 4. Vitamin D Deficiency and Insufficiency

Because of unregulated hydroxylation by the liver, 25-hydroxyvitamin D is used as the marker of vitamin D status. Low levels of serum vitamin D (25-hydroxyvitamin D) may define vitamin D deficiency versus insufficiency [12]. However, the definitions of low levels of vitamin D vary among authors and nutritional societies/authorities. For some authors, vitamin D deficiency is the level below which osteomalacia may appear [13]. In general, this occurs at a concentration below 25 nmol/L [12]. Vitamin D insufficiency may be defined as the level below which PTH begins to rise. Depending on the studies, this level may vary from 25 to 75 nmol/L. Another definition is only based on serum concentration thresholds without considering biological or clinical abnormalities [14]. According to this definition, vitamin D status may be divided into 4 group levels: severe deficiency defined by a concentration of less than 27.5 nmol/L, deficiency between 27.5 and 49.9 nmol/L, insufficiency between 50 and 75 nmol/L, and optimal above 75 nmol/L [15, 16]. Differences of definitions between authors may explain, at least in part, conflicting resulting from meta-analysis addressing vitamin D status and outcomes. Vitamin D deficiency/insufficiency is highly prevalent but the magnitude of hypovitaminosis D may vary depending on the population studied and regional and seasonal considerations. We will only comment on examples of prevalence in the elderly population. Among healthy elderly patients in Argentina, Oliveri et al. found prevalence between 52% and 87% of vitamin D insufficiency (serum level below 50 nmol/L) in the winter depending on the latitude [17]. In another study addressing institutionalized elderly patients from Buenos Aires, 41% of the residents had a vitamin D serum level of less than 25 nmol/L [10]. In a study looking at elderly Italian women, the prevalence of low vitamin D, defined as a serum level below 30 nmol/L, was 51% in the December–May period and 17% in the June–November period [18].

## 5. Vitamin D and the Skeletal Muscle: Molecular Pathways

**Figure 1:**
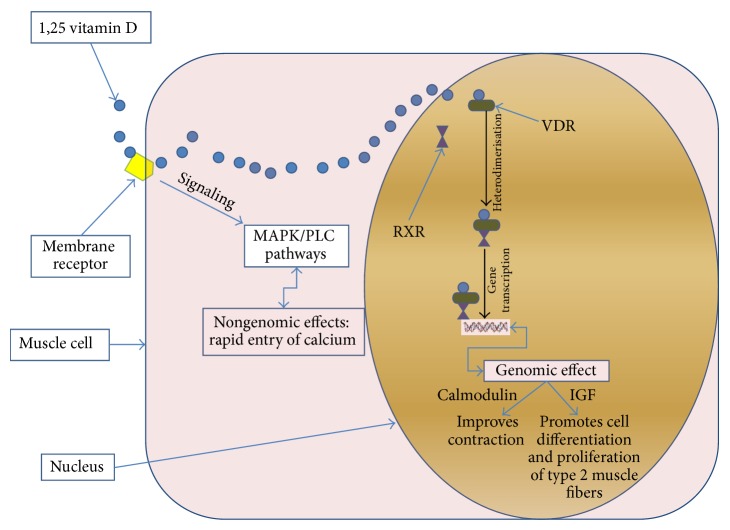
Effects of 1,25 vitamin D on muscle cells: molecular and nuclear pathways. 1,25 vitamin D binds the vitamin D receptor (VDR) in the nucleus, where it forms a complex with the retinoid receptor (RXR). The complex 1,25 vitamin D/VDR/XDR activates gene transcription, leading to known genomic effects. On the other hand, 1,25 vitamin D binds a putative membrane receptor which activates MAP kinase (MAPK) and phospholipase C (PLC) pathways leading to nongenomic effects.

Vitamin D receptors (VDR) are expressed in a large number of human cell types, including skeletal muscle cells, indicating the potential for widespread effects [2, 19, 20]. Two mechanisms by which vitamin D may act in skeletal muscle have been proposed (Figure 1). VDR acts as a nuclear receptor which mediates the so-called genomic effects; VDR also acts via nonnuclear receptor mediating nongenomic actions. Genomic effects have been well characterized in studies* in vitro* [8, 21]. VDR is a ligand-dependent transcription factor, which belongs to the steroid-thyroid hormone receptor gene superfamily. Once transported in the nucleus by an intracellular binding protein, calcitriol binds to its nuclear receptor which results in gene transcription and subsequent* de novo* protein synthesis. At the nuclear level, the activation of VDR induces heterodimerization between the active VDR and the retinoic receptor (RXR). This leads to the activation of the vitamin D response element (VDRE), a complex of genes coding for the “genomic effects” of vitamin D. Genomic effects of VDR include the increase in calcium handling by enhancing the activities of the calcium binding protein (calbindin-D9K) in cell sarcoplasm [22, 23], muscle cell differentiation and proliferation through effects on insulin growth factor expression which in turn induces skeletal muscle hypertrophy [24]. Mechanisms leading to vitamin D nongenomic effects are not definitely elucidated. 1,25 vitamin D appears to bind a membrane receptor which activates a transduction signal inducing MAP kinase (MAPK) and phospholipase C (PLC) pathways, which in turn lead to a rapid influx of calcium into the cell [8, 21]. The origin of this membrane receptor is controversial. Some authors claimed that this membrane receptor is the intranuclear VDR receptor itself, which translocates from the nucleus to the plasma membrane, while others suggest that this is a distinct receptor [25, 26]. Some studies have explored vitamin D molecular pathways and VDR, through vitamin D supplementation. For instance, Ceglia et al. showed that, in elderly women, a supplementation of vitamin D (4000 IU/day) during 4 months was associated with a 30% increase in intramyonuclear VDR concentration and a 10% increase in muscle fiber cross-sectional area, especially type 2 fibers [27]. The VDR has several polymorphisms, some of which may have clinical significance. Geusens et al. showed that the presence (allele bb) or absence (allele BB) of a restriction fragment (BsmI) may determine muscle strength; that is, subjects with the bb phenotype had 23% higher muscle strength in the quadriceps than those with the BB phenotype [28].

## 6. Hypovitaminosis D and Physical Performance: Observational Studies

Many observational studies have investigated clinical relationships between vitamin D serum concentration and physical performance. In the Invecchiare in Chianti (InCHIANTI) study (966 individuals, 435 men and 531 women) with a mean age of 75 years, a significant association between low levels of vitamin D and poor physical performance as assessed by the handgrip strength test and a short physical performance battery test (ability to stand from a chair and ability to maintain balance in progressively more challenging positions) was found [29]. Individuals with serum vitamin D below 25 nmol/L performed lower than those with a level above 25 nmol/L. Muscle strength using a handgrip test was also significantly greater in subjects with vitamin D levels higher than 50 nmol/L than in those with levels below this threshold [29]. Mastaglia et al. reported that, in healthy women aged over 65 years (*n* = 54), vitamin D levels above 50 nmol/L were associated with a higher muscle strength from lower limbs (stronger knee extensor of 13.4 ± 2.7 versus 11.6 ± 2.5 kg, *P* < 0.03) [30]. Zamboni et al. measured serum vitamin D in elderly women (*n* = 175) and men (*n* = 94) and disability was self-reported using different questionnaires. Individuals reporting disability had a lower serum vitamin D level than those without self-reported disability [31]. This observation was confirmed in the Longitudinal Aging Study Amsterdam (LASA) prospective study which followed 1200 elderly men and women (600 men and 634 women) during 3 years. A physical assessment was performed at baseline and after 3 years. Subjects with vitamin D serum levels below 25 nmol/L had a greater chance of showing a decline in physical performance, defined by a change in the Edwards-Nunnally Index, than those with levels higher than 75 nmol/L (OR: 2.21, 95% CI 1.00–4.87) [32]. Using data from the same cohort, Visser et al. demonstrated that elderly individuals with a low vitamin D level (<25 nmol/L) had a 2.5-fold increase in the risk of developing sarcopenia, defined as a loss of handgrip strength of more than 40% or a loss of muscle mass of more than 3%, in a 3-year time period, compared with those with levels of > 50 nmol/L [33]. In a study investigating elderly patients with falls (230 men and 370 women), a higher serum concentration of vitamin D was associated with a 3 times faster “time and get up” (TUG) test (i.e., the time required for the patient to stand up from a standard chair, walk a distance of 3 meters, turn around, walk back to the chair, and sit down again), with a five times* faster* “sit to stand” test in men, and with a 2.5 times faster TUG test in women. The data suggest possible differences in the effects of vitamin D according to the gender [34]. However, in the Progetto Veneto Anziani (Pro.V.A) study, which included 2694 community-dwelling elderly patients (1597 females and 1097 males, mean age of 74 years, 40% of women and 20% of men with a serum vitamin D below 50 nmol/L), it was shown that lower vitamin D levels were associated with a lower 6-minute walking test and weaker strength, independently of gender [35]. Positive effects of vitamin D reserves are not only observed in older persons. In a study including 1000 healthy European adolescents (470 males and 530 females), handgrip test performance was positively associated with vitamin D levels in females [3]. In another study including young women (age between 19 and 29 years), there was a correlation between vitamin D level and the handgrip test in both dominant and nondominant arms [36]. An additional study in healthy men (*n* = 205) and women (*n* = 214) (mean age of 43 years) showed a relationship between vitamin D and isometric/isokinetic arm strengths in multivariate analysis [37]. In the particular setting of CKD, 3 small studies, one study in CKD treated conservatively and 2 studies in dialysis patients, suggest a positive relationship between vitamin D status and functional ability [38–40].

A few studies did not confirm an association between vitamin D status and physical performances. Pramyothin et al. performed a study in older Hawaii women of Japanese ancestry, a population known for its very low rate of falls, a high dietary intake in vitamin D, and a large exposure to sunlight [41]. In this population, mean vitamin D level was 80 nmol/L and no subject presented vitamin D deficiency. There was no relationship between vitamin D level, physical strength test (except for the quadriceps), falls, and daily activities. The authors conclude that the absence of relationship was due to the very high level of vitamin D at baseline. In addition, another study in young men (mean age of 47 years) from Ceglia et al. including more than 1000 individuals did not demonstrate a link between vitamin D levels and physical performances [42]. However, only 20% of the subjects had a vitamin D level below 50 nmol/L [42]. These 2 negative studies, along with other observations, suggest the existence of a threshold in vitamin D level, below which hypovitaminosis D may negatively affect muscle function [29, 30]. However, in one study including 367 elderly individuals aged more than 80 years with 80% prevalence in vitamin D insufficiency, there was again no relationship between vitamin D level and physical performance, as assessed by gait speed, hand grip test, and a static balance test. In this case, the authors explain the absence of association by the decrease in VDR expression observed in very old individuals [43].

To sum up, most of the observational studies report a significant association between hypovitaminosis D and muscle dysfunction in all categories of ages, except in very old individuals. On the contrary, vitamin D levels greater than 50 nmol/L are associated with the lowest probability of muscle dysfunction. Some studies suggest that gender may influence the association between vitamin D and skeletal muscle function.

## 7. Can Vitamin D Supplementation Improve Muscle Function?

Several RCTs and meta-analyses have investigated the effect of vitamin D supplementation on muscle function. Two RCTs in Asians, one in healthy young volunteers [44] and another one in elderly women, compared the supplementation of daily or weekly (Table 1) vitamin D combined with calcium versus calcium alone or placebo [11]. These studies reported a benefit of vitamin D supplementation during 3 to 6 months on muscle function, that is, an improvement in quadriceps strength as measured by an isokinetic dynamometer device [11] and an improvement in 6 min walk test [44]. Another trial in 300 elderly women with a level of vitamin D <60 nmol/L demonstrated a benefit with a daily supplementation of 2000 IU of vitamin D on the TUG test. The subjects from the lowest quartile had an additional improvement in muscular strength [45]. Benefits of vitamin D supplementation were also shown in teenager females. Ward et al. randomized 69 postmenarchal females to receive either 4 doses of 150,000 IU of vitamin D2 or placebo, over one year. In the interventional group, mean vitamin D level was greater than 50 nmol/L and this was associated with improved jump velocity [46]. Another small interventional trial including young elite ballet dancers (11 males and 13 females, mean age: 28) showed an improved isometric strength and less injuries in the group receiving a daily dose of 2000 IU vitamin D [47]. In contrast, 2 trials did not show a benefit of vitamin D supplementation [48, 49]. However, one of these studies included healthy men, with very good physical performance at baseline and without vitamin D deficiency/insufficiency [48]. In the other study, vitamin D was administrated at a lower dose, that is, 8400 IU of vitamin D3 once weekly, and this failed to improve physical performance, but in the latter study, there was a positive effect on balance in a subgroup of patients with markedly low balance at baseline [49]. Two additional RCTs using intermittent large amounts of vitamin D did not show a benefit on physical performance [50–53]. One study tested the effect of 150,000 IU of cholecalciferol every 3 months in 689 elderly women (mean age above 76 years) for 9 months. Muscle strength was assessed at baseline and every 3 months with a dynamometer, and mobility was measured by a TUG test [52]. There was no difference between the treatment and the control groups. However, vitamin D was measured in only 40 subjects of the 700 subjects with indeed a high baseline level (65.8 nmol/L). In the other study, 173 young healthy females (mean age of 21 years) were given a supplementation of 60,000 IU of cholecalciferol, once a week for 8 weeks, and then 60,000 IU every 2 months for 4 months. There was no difference in muscle strength at 6 months. These findings suggest that intermittent high doses of vitamin D may not be effective at improving muscular strength. This lack of clinical effect may be explained by the inability of high intermittent doses of vitamin D to maintain high serum levels for a sustained period, as suggested by Gupta et al. [44].

The meta-analysis from Muir and Montero-Odasso, which pooled results from 13 RCTs in individuals older than 60 years old, supported a small benefit of daily vitamin D supplementation (800 IU to 1000 IU per day) for muscle strength and balance [54]. However, another meta-analysis by Stockton et al. looking at 17 RCTs in individuals of all ages including younger subjects only showed a benefit in muscle strength in subjects with vitamin D serum levels below 25 nmol/L at baseline [55].

Whether 1,25 vitamin D is also effective to improve muscle function has been insufficiently investigated. A prospective uncontrolled trial, not meeting the criteria of our search but including 2000 elderly subjects (mean age of 75 years, 80% females), showed that a daily supplementation of 1 mcg of alfacalcidol leads to significant improvements of the TUG, chair rising test, and tandem gait tests, used as surrogates of muscle performance and risk of fall [56].

Overall, data from RCT and meta-analysis support a positive effect of daily vitamin D supplementation on muscle function, especially in older individuals with vitamin D insufficiency/deficiency at baseline. A daily dose of 1000 UI appears to be sufficient to obtain significant improvements. In contrast, large intermittent doses of vitamin D do not appear to be efficient at improving muscle strength.

## 8. Relationship between Vitamin D Status, Muscle, and Falls

The known association of vitamin D insufficiency and increased risk of falls and fractures in the elderly [50, 51, 57–59] was thought to depend on bone remodeling via the rise of PTH [60]. However, current understanding has highlighted the importance of a direct effect of vitamin D on muscle strength and function [21] to explain this association. Because vitamin D has an effect on type 2 muscle fibers, it was tempting to speculate a protective effect of vitamin D on falls, via improvement in muscle function. A high number of RCTs have investigated whether vitamin D supplementation had an effect on muscle function and the incidence of falls. A Cochrane review published in 2003 [61] evaluated the efficacy of supplementation with vitamin D analogs, either alone or with calcium as cosupplementation at preventing falls. The overall analysis of vitamin D versus control found no significant difference in the rate of falls when applied to unselected community-dwelling and hospitalized elderly subjects (RR 0.87, 95% CI 0.70–1.08). In contrast, in more recent studies, benefits of vitamin D supplementation were significant. Pfeifer et al. demonstrated a reduction in falls of 27 and 39% at one year and 20 months, respectively, in community-dwelling seniors supplemented with 800 IU vitamin D and calcium daily versus with calcium only [62]. This reduction in falls was correlated with an improvement in quadriceps strength and an improvement in the TUG test. These results are consistent with the study of Bischoff et al. who showed a 49% reduction of falls in elderly women from a geriatric ward supplemented with 800 IU per day of vitamin D [63]. In the same period, results from 3 meta-analyses demonstrate a significant reduction in the odd ratio of falls in individuals supplemented with vitamin D [64–66].

**Figure 2:**
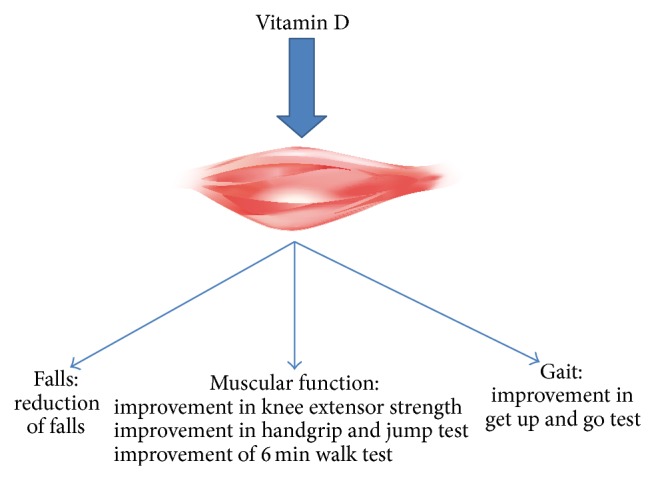
Clinical effects of vitamin D on muscles gait and falls.

Figure 2 shows the effects of vitamin D on muscle function, gait, and falls. Table 1 summarizes the studies which investigated the clinical effects of vitamin D on muscle strength, function, and the risk of falls. The most recent report of the Endocrine Society Clinical Practice Guidelines recommends vitamin D supplementation depending on age and clinical circumstances, in particular in order to prevent falls in populations at risk [15]. However, a recent paper from the Institute of Medicine from the United States questioned the effect of vitamin D supplementation on extraskeletal outcomes [67], in particular in the setting of falls, arguing that the meta-analysis of Bischoff-Ferrari et al. provided misleading conclusions, that is, a vitamin D-associated decrease of 22% in the risk of falls, due to heterogeneity considerations in the 5 RCTs considered [66].

## 9. A Link between Vitamin D and Frailty?

The term “frailty” is becoming more and more popular in geriatric medicine. However, its definition is vague. The Oxford dictionary defined it by “the condition of being weak and delicate.” A more precise definition is given by Fried who defined frailty as “a biologic syndrome of decrease reserve and resistance to stressors that results from cumulative declines across multiple physiologic systems and causes vulnerability to adverse outcomes [68].” Criteria of the frail phenotype have been described in order to translate the above theoretical definition into clinical indicators [68]. These are as follows: unintentional weight loss, self-reported exhaustion, weakness (grip strength), slow walking speed, and low physical activity. According to these clinical criteria, 3 phenotypes have been identified: robust: 0 criteria; prefrail: between 1 and 2 criteria; frail: 3 or more criteria. The majority of these criteria are related to locomotion and physical strength. Thus, it looks readily conceivable that hypovitaminosis D may lead to frailty, through negative effects on muscle strength and/or function.

The association between vitamin D status and frailty has been studied in a number of observational studies. Data from an observational study from Hirani et al. which included 1659 community-dwelling men, with a 10% prevalence of frailty, showed that low vitamin D levels were independently associated with frailty [69]. A similar association was found by Tajar et al. in another cohort of elderly men. Subjects with vitamin D levels <50 nmol/L had an odd ratio of 2,37 of being classified into the “frail” versus the “robust” phenotype [70]. Using data from the third National Health and Nutrition Survey (NHANES), Wilhelm-Leen et al. found an association between frailty and a low vitamin D status in both elderly men and women, with overall 4-fold increase in the odd ratio of frailty [71]. Vitamin D not only is associated with frailty but also appears to be associated with an increased risk to develop frailty over time in women. In a prospective study including elderly women (age > 69 years), nonfrail women at baseline but displaying a vitamin D level of less than 50 nmol/L had a higher risk of becoming frail during the 4.5 years of follow-up than women with a higher level of vitamin D [72]. In a study from patients with cardiac insufficiency, Boxer et al. found an association between low vitamin D levels and the frail phenotype. In particular, vitamin D levels and the result of the 6-minute walking test were correlated [73]. In cardiac diseases, this functional test is known to predict survival [74]. Thus, low vitamin D is hypothesized to link with mortality in this setting. A prospective study including 4000 individuals (1943 men and 2788 women, mean age: 70), followed up to 12 years, indeed found a link between lower levels of vitamin D, frailty, and mortality. An assessment of vitamin D status and the physical phenotype (robust/prefrail/frail) were performed at baseline [75].

Mortality was positively associated with frailty. Frail individuals with a low vitamin D level were at increased risk (hazard ratio of 2.98) of death during the follow-up compared to robust individuals with a high level of vitamin D. Thus, overall, a clear association between vitamin D level and frailty has been demonstrated. Furthermore, interplays between vitamin D status, frailty, and mortality appear plausible. Whether vitamin D supplementation in frail subjects may reduce mortality is challenging and needs to be investigated in the future.

## 10. Conclusion

Consistent relationships exist between vitamin D status and muscle function, especially in the elderly frail patient. There is evidence that hypovitaminosis D is associated with a decline in muscle function. Vitamin D supplementation has beneficial effects on muscle strength, balance, and gait in diverse settings including adolescents, the elderly, and CKD patients. However, the effects of vitamin D on the prevention of falls are still a matter of debate due to conflicting interpretation of data. Differences in the dose of supplementation, type of vitamin D, and discrepancies in the threshold to define vitamin D deficiency/insufficiency may partly explain these disagreements. A low vitamin D status is consistently associated with frailty. Considering that vitamin D supplementation is safe and inexpensive, it is worthy to recommend vitamin D supplementation in patients at risk for falls, such as elderly patients, nursing home residents, frail patients with gait and balance and visual impairments, and patients with chronic diseases. These patients are most likely to have low levels of vitamin D and muscle loss/dysfunction, thus justifying supplementation independent of a putative effect on the prevention of falls.

## Figures and Tables

**Table 1:** Summary of RCT and meta-analysis regarding effects of vitamin D on muscle function and falls.

Type of study	Author	Number of subjects	Type of subjects	Mean age (years)	Intervention	Duration	Results
RCT	Kenny et al. (2003) [48]	65	Healthy men	76	1,000 IU/d vitamin D versus placebo	6 months	No increase in muscle strength or improvement in physical performance.

RCT	Songpatanasilp et al. (2009) [11]	72	Postmenopausal females	70	Ca 1500 mg/d + alfacalcidol 0.5 ug/d versus Ca 1500 mg/d	12 weeks	Improvement in muscular strength.

RCT	Lips et al. (2010) [49]	226	Elderly males and females with vitamin D <50 nmol/L	77	8400 IU/week vitamin D versus placebo	16 weeks	Improvement of balance in a subgroup with severe balance impairment at baseline.

RCT	Ward et al. (2010) [46]	69	Postmenarchal females (12 to 14 years old) with vitamin D <25 nmol/L	13	4 doses of 150,000 IU vitamin D every 3 months versus placebo	12 months	Increase in jump velocity in girls with low vitamin D levels. No improvement in strength in others.

RCT	Gupta et al. (2010) [44]	40	Healthy males and females	31	60,000 IU/week for 8 weeks followed by 60,000 IU/month for 4 months vitamin D + 1000 mg Ca/d versus placebo	6 months	Enhanced skeletal muscle strength and physical performance.

RCT	Zhu et al. (2010) [45]	300	Elderly females with vitamin D <60 nmol/L	77	1,000 IU/d vitamin D + Ca 1000 mg/d versus Ca 1000 mg/d + placebo	12 months	Enhanced skeletal muscle strength and physical performance in patient with the lowest vitamin D level.

RCT	Taskapan et al. (2011) [39]	25 (CKD stages 3-4) 47 (PD)	CKD and PD with vitamin D <50 nmol/L	NA	50000 IU/week vitamin D	4 to 8 weeks	Improvement in physical performance tests.

UCT	Schacht and Ringe (2012) [56]	2100	Males and postmenopausal females	75	1 mcg/d calciferol	6 months	Improved physical performance.

RCT	Glendenning et al. (2012) [52]	690	Elderly females (age >70)	77	150,000 IU/every 3 months vitamin D versus placebo	9 months	No differences in falls and physical performance between the groups.

RCT	Goswami et al. (2012) [53]	173	Healthy females	22	60,000 IU/week every 8 weeks then 60, 000 IU/fortnight + Ca 500 mg/d versus 60,000 IU/week every 8 weeks then 60, 000 IU/fortnight + placebo versus Ca 500 mg/d + placebo versus placebo	6 months	No differences in muscle strength between the groups.

RCT	Ceglia and Harris (2013) [21, 27]	21	Females with limited mobility	78	4000 IU/d vitamin D	4 months	Increase of intramyonuclear VDR concentration. Increase in muscle fibers.

RCT	Wyon et al. (2014) [47]	24	Elite ballet dancers	28	2000 IU/d vitamin D versus placebo	4 months	Increased muscle performance and less injury.

Meta	Muir and Montero-Odasso (2011) [54]	2268 (13 RCTs)	Elderly males and females (age >65)	78	Vitamin D supplementation		Beneficial effects on strength and balance.

Meta	Stockton et al. (2011) [55]	5072 (17 RCTs)	Males and females of all ages	NA	Vitamin D supplementation		Increase in muscle strength in adults with baseline vitamin D <25 nmol/L.

RCT	Bischoff et al. (2003) [63]	122	Elderly females	85	Ca 1200 mg and 800 IU/d vitamin D versus Ca 1200 mg/d	12 weeks	Reduced risk of fall.

RCT	Pfeifer et al. (2009) [62]	242	Community-dwelling elderly males and females	77	800 IU/d vitamin D + Ca 1000 mg/d versus Ca 1000 mg/d	12 months	Reduced number of falls and improvement in muscle function.

Meta	Gillespie (2003)	461 (3 RCTs)	Elderly males and females	NA	Vitamin D supplementation		No reduction in the risk of fall.

Meta	Bischoff-Ferrari et al. (2004) [66]	10001 (10 RCTs with sensitivity analysis) 1237 (5 RCTs without sensitivity analysis)	Elderly males and females, age >65	70	Vitamin D supplementation		Reduced risk of fall.

Meta	Michael et al. [64] (2010)	5809 (9 RCTs)	Elderly males and females	NA	Vitamin D supplementation		Reduced risk of fall.

Meta	Murad et al. (2011) [65]	45782 (26 RCTs)	Males and females (all ages)	NA	Vitamin D + Ca supplementation		Reduced risk of fall.

RCT: randomized control trial, UCT: uncontrolled trial,Meta: meta-analysis, NA: nonavailable, CKD: chronic kidney disease, PD: peritoneal dialysis, VDR: vitamin D receptor, and Ca: calcium.
